# Spatial and Temporal Variation in PM_2.5_ Chemical Composition in the United States for Health Effects Studies

**DOI:** 10.1289/ehp.9621

**Published:** 2007-04-20

**Authors:** Michelle L. Bell, Francesca Dominici, Keita Ebisu, Scott L. Zeger, Jonathan M. Samet

**Affiliations:** 1 School of Forestry and Environmental Studies, Yale University, New Haven, Connecticut, USA; 2 Department of Biostatistics, Johns Hopkins Bloomberg School of Public Health, Baltimore, Maryland, USA; 3 School of Public Health, Yale University, New Haven, Connecticut, USA; 4 Department of Epidemiology, Johns Hopkins Bloomberg School of Public Health, Baltimore, Maryland, USA

**Keywords:** elemental carbon, organic carbon, particulate matter, PM_2.5_, nitrate, sulfate

## Abstract

**Background:**

Although numerous studies have demonstrated links between particulate matter (PM) and adverse health effects, the chemical components of the PM mixture that cause injury are unknown.

**Objectives:**

This work characterizes spatial and temporal variability of PM_2.5_ (PM with aerodynamic diameter < 2.5 μm) components in the United States; our objective is to identify components for assessment in epidemiologic studies.

**Methods:**

We constructed a database of 52 PM_2.5_ component concentrations for 187 U.S. counties for 2000–2005. First, we describe the challenges inherent to analysis of a national PM_2.5_ chemical composition database. Second, we identify components that contribute substantially to and/or co-vary with PM_2.5_ total mass. Third, we characterize the seasonal and regional variability of targeted components.

**Results:**

Strong seasonal and geographic variations in PM_2.5_ chemical composition are identified. Only seven of the 52 components contributed ≥ 1% to total mass for yearly or seasonal averages [ammonium (NH_4_^+^), elemental carbon (EC), organic carbon matter (OCM), nitrate (NO_3_^−^), silicon, sodium (Na^+^), and sulfate (SO_4_^2−^)]. Strongest correlations with PM_2.5_ total mass were with NH_4_^+^ (yearly), OCM (especially winter), NO_3_^−^ (winter), and SO_4_^2−^ (yearly, spring, autumn, and summer), with particularly strong correlations for NH_4_^+^ and SO_4_^2−^ in summer. Components that co-varied with PM_2.5_ total mass, based on daily detrended data, were NH_4_^+^, SO_4_^2−^_,_ OCM, NO_3_^2−^, bromine, and EC.

**Conclusions:**

The subset of identified PM_2.5_ components should be investigated further to determine whether their daily variation is associated with daily variation of health indicators, and whether their seasonal and regional patterns can explain the seasonal and regional heterogeneity in PM_10_ (PM with aerodynamic diameter < 10 μm) and PM_2.5_ health risks.

Numerous studies have shown that airborne particulate matter (PM) is associated with adverse health effects, including increased risk of premature mortality, hospital admissions, and higher rates of adverse respiratory health indicators in children [Pope and Dockery 2006; U.S. Environmental Protection Agency (EPA) 2006]. Although the health effects of airborne particles have been investigated vigorously for decades, uncertainty persists concerning those characteristics of PM that determine toxicity. To date, studies on the health impacts of PM exposure have used a variety of metrics for PM, including total suspended particles (TSP), coefficient of haze (COH), black smoke, British smoke, KM (a measure of particulate optical reflectance), and PM_10_ and PM_2.5_ (PM with an aerodynamic diameter of < 10 μm and < 2.5 μm, respectively). These indicators reflect PM mass in particular size ranges but not composition specifically. For effective control of particle pollution, information is needed on which sources contribute to the PM characteristics associated with health risk

A growing number of studies have investigated the health effects of PM_2.5_, a PM indicator incorporated in the 1997 National Ambient Air Quality Standards (NAAQS) (e.g., Dominici et al. 2006; Franklin et al. 2007; Laden et al. 2006; Schwartz et al. 2002). This indicator was selected in the 1997 NAAQS because of well-established knowledge of the dosimetry of particles in this size range within the respiratory tract and epidemiologic evidence indicating adverse effects of PM_2.5_ specifically. However, lacking evidence on the characteristics of PM in this size range that may determine toxicity, a general mass-based standard was promulgated. Characteristics of PM_2.5_ that may be relevant to toxicity include metals, organic compounds adsorbed onto particles or forming particles themselves, biologic components, sulfate (SO_4_^2−^), nitrate (NO_3_^−^), acidity, and surface-adsorbed reactive gases such as ozone (O_3_) [Health Effects Institute 2002; National Research Council (NRC) 2004]. Studies have associated several chemical components of PM_2.5_ with mortality including iron (Fe), nickel (Ni), zinc (Zn) (Burnett et al. 2000), ammonium nitrate (Fairley 1999), elemental carbon (EC), organic carbon (OC), nitrates (Ostro et al. 2007), and sulfates (Burnett et al. 2000; Ostro 1995).

Recognizing the need for further research on PM characteristics and health, the U.S. EPA has established a national monitoring network for PM_2.5_ that provides data on the chemical composition of PM (U.S. EPA 2006). As the data accumulate, they will foster epidemiologic studies designed to assess health risks associated with spatial and temporal variation in PM characteristics. In this article we report analyses of a database constructed from the U.S. EPA monitoring results for 52 PM_2.5_ components in 187 counties in the continental United States for the period 2000–2005. We describe the spatial and temporal patterns of variation of PM_2.5_ chemical components and identify components that might be evaluated in studies of PM_2.5_ and human health effects.

For an individual chemical constituent to be a mediator of the risk associated with PM_2.5_ total mass, the concentration of that component must co-vary with the more general mass variable used in epidemiologic research (i.e., PM_2.5_ total mass); however, we recognize that multiple components may contribute to the risk and that components may interact. Other components that may be harmful to human health may not be related to the observed relationships between PM_2.5_ and health. We provide descriptive analyses intended to identify candidate PM_2.5_ components that meet this criterion of being correlated with PM_2.5_ total mass and to summarize the spatial and temporal variation of such components. These candidate components should be explored further to determine whether they mediate the effect of PM_2.5_ total mass and to investigate the underlying biological mechanism.

## Methods

### Database development

We developed a database of concentrations for 52 PM_2.5_ components and PM_2.5_ total mass for 187 continental U.S. counties for the period February 2000 to December 2005, based on data obtained from the U.S. EPA’s Office of Air Quality Planning and Standards (U.S. EPA 2006). Counties and components are listed in the Tables S1 and S2 of the Supplemental Material (http://www.ehponline.org/docs/2007/9621/suppl.pdf). Not all counties had the full complement of data for the entire time period. Although most monitors provide data every 6 days, the average frequency of measurement by monitor ranged from 3.1 to 11.9 days. We generated countywide estimates for each PM_2.5_ component based on an analysis of the monitor or monitors within each county.

In developing and analyzing the data set, we needed to address several key issues, described below, for which we developed a protocol to combine the data to generate a countywide average.

#### Suspect data

The U.S. EPA coded some observations as problematic or unusual (e.g., “lab issues”). These individual observations, which included many extreme values, were omitted.

#### Noncontinental counties

We omitted nonmainland counties—that is, those in Hawaii and Alaska.

#### Co-location of monitors

Fifteen of 259 U.S. EPA monitoring sites (5.8%) had multiple monitors for duplicate sampling on the same day. Data from multiple monitors in the same site were treated as repeat measurements at the same site and were averaged to generate an overall observation at that location. After values from co-located monitors were averaged, county-level exposures were estimated as the average across monitors within the county.

#### Counties with little data

We omitted counties with data collected on PM_2.5_ total mass or for any of the individual PM_2.5_ components for only a brief period (< 6 months or < 30 observations). A total of 11.4% of the individual counties (2.7% of observation days) were omitted for this reason.

#### Check of unusual values

Observation days were omitted if the highest PM_2.5_ value recorded was over three times higher than the second highest value. This criterion excluded only two observation days.

OC measurements require adjustment to correct for field blanks and to account for elements such as oxygen and hydrogen that are associated with OC to estimate organic matter. Organic carbon matter (*OCM)* was calculated as


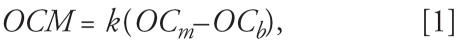


where *OCM* = organic carbon matter; *k* = adjustment factor to account for noncarbon organic matter (1.4); *OC_m_* = measured organic carbon; *OC_b_* = organic carbon for blank filters. Blank filter correction values were based on U.S. EPA data (U.S. EPA 2006). Recent analysis has shown that OC blank values from 2001 to 2005 have increased for some samplers and decreased for others (Frank NH, unpublished data). We performed a sensitivity analysis using a data set with *OCM* estimated by sampler- and year-specific blank correction values (Frank NH, unpublished data). This alternative value for *OCM* is specified as *OCM2.*

### Analysis

First we determined which PM_2.5_ components contributed a substantial fraction to total PM_2.5_ mass, for either the yearly average or any seasonal average. Seasons were defined based on 3-month periods (e.g., summer was defined as June–August). Second, we identified the components that co-vary day to day with total PM_2.5_ mass. We calculated yearly and seasonal correlations between PM_2.5_ total mass and each component and with the corresponding seasonally detrended time series,


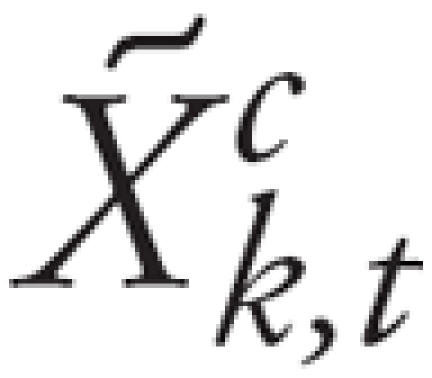


, defined as:


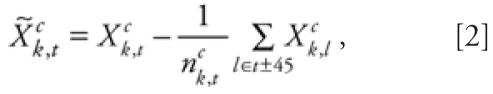


where *X^c^_k,t_* = the concentration of component *k* at time *t* for county *c*,


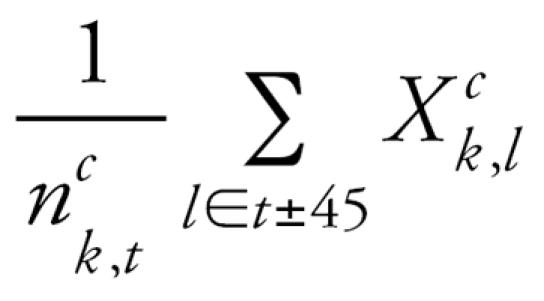


is the 91-day moving average of the concentration of component *k* for county *c* centered at time *t*, and *n^c^_k,t_* = the number of days with observations for component *k* for county *c* for a 91-day moving average centered at time *t*.

Analysis of detrended data included only counties with more than one full year of observations.

## Results

**Figure 1:**
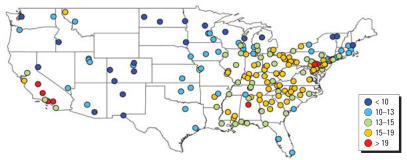
PM_2.5_ average (μg/m^3^) for 187 U.S. counties, 2000–2005.

**Figure 2:**
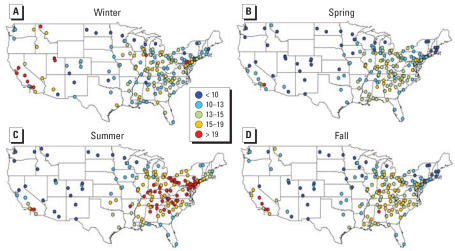
Seasonal PM_2.5_ averages (μg/m^3^) for 187 U.S. counties, 2000–2005.

The original data set included 62,690 observation days across all sites (i.e., monitor-days of data), which dropped to 48,591 observations for 187 counties with the exclusions described earlier. Many counties had data for only a portion of the study period. The average number of observations per county for PM_2.5_ total mass was 260 days (range, 41–676). Figure S1 in the Supplemental Material (http://www.ehponline.org/docs/2007/9621/suppl.pdf) shows the number of PM_2.5_ observations per county for the study period. For other components, the average number of observations per county ranged from 248 days [sodium ion (Na^+^)] to 260 days (OC). Every county had data available for every season, with 26.3% of the PM_2.5_ total mass data from summer, 26.5% from autumn, 22.7% from winter, and 24.6% from spring. Figure 1 shows average PM_2.5_ levels for the study period (2000–2005) for each county. Overall, PM_2.5_ levels were higher in the eastern United States and California, and lowest in the central regions and Northwest. However, PM_2.5_ concentrations had strong seasonal patterns that differed by region (Figure 2). On the west coast, levels peaked in winter and autumn, especially for northern California, whereas on the east coast higher levels were recorded for summer. Concentrations remained lower in the central United States throughout the year.

Table 1 provides summary statistics for each PM_2.5_ component for the full year, and for summer and winter. Many components show strong seasonal patterns. For example, NO_3_^−^, chlorine (Cl), Zn, Ni, and bromine (Br) are 3.6, 3.2, 1.5, 1.4, and 1.4 times higher in winter than in summer, respectively. Aluminum (Al), titanium (Ti), magnesium (Mg), silicon (Si), and SO_4_^2−^ were 1.5 to > 2 times higher in summer than in winter. Other components did not show distinct seasonal patterns. The results in Table 1 and other summary measures that provide a national average conceal spatial heterogeneity on smaller spatial domains. Similarly, presentations of yearly values obscure seasonal differences.

**Figure 3:**
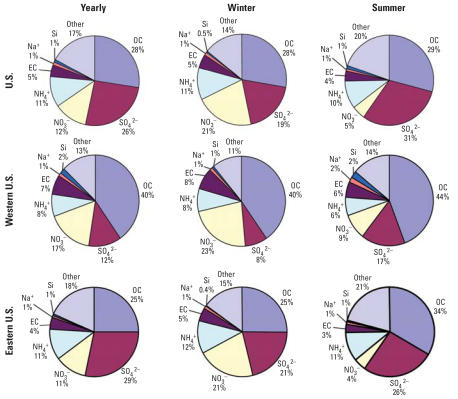
Percent of PM_2.5_ composition by component for yearly, winter, and summer averages, by region.

If a PM_2.5_ component contributes to the associations with risks for health outcomes observed in time-series studies based on PM_2.5_ total mass, the component would be expected to exhibit strong day-to-day variation with PM_2.5_ total mass. Many such components are likely to contribute substantially to PM_2.5_ total mass. We first identified components that comprise the majority of overall PM_2.5_ mass. Only 7 of the 52 components contributed ≥ 1% to the PM_2.5_ total mass for the yearly average or any of the seasonal averages across all 187 counties. Those components (NH_4_^+^, EC, OCM, NO_3_^−^, Si, Na^+^, and SO_4_^2−^) comprised 79–85% of the total PM_2.5_ mass for the yearly or seasonal averages. Figure 3 shows the percentages of PM_2.5_ contributed by these components for yearly, winter, and summer averages, for nationwide, eastern U.S., and western U.S. averages. SO_4_^2−^ is a larger contributor in summer, whereas NO_3_^−^ is a larger contributor in winter for both regions. Although Figure 3 presents results for the two U.S. regions, spatial heterogeneity can also exist within regions. Further, this analysis is limited to the components included in the database, and other components or chemical forms (e.g., ferric oxide) that were not measured could also have contributed ≥ 1% to total PM_2.5_ mass.

We also examined if any components contributed 1% or more to PM_2.5_ within any individual county for either a yearly or seasonal average. Components meeting this criteria were Al, calcium (Ca), Cl, Fe, and potassium (K), which on average provide 0.18–0.62% of PM_2.5_ total mass across the whole year, but in some cities contributed up to 5.4% for a given season. The contribution of these components to PM_2.5_ total mass on average across all communities and the minimum and maximum values for any single community are provided in the Supplemental Material (Table S3; http://www.ehponline.org/docs/2007/9621/suppl.pdf) for yearly and seasonal averages.

**Figure 4:**
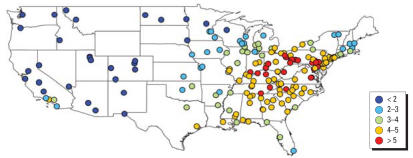
Sulfate PM_2.5_ (μg/m^3^) averages for 187 U.S. counties, 2000–2005.

**Figure 5:**
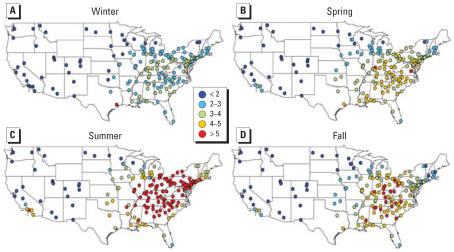
Seasonal sulfate PM_2.5_ (μg/m^3^) averages for 187 U.S. counties, 2000–2005.

**Figure 6:**
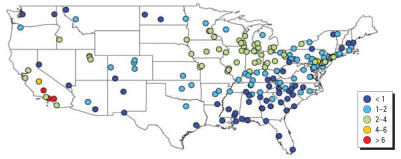
Nitrate PM_2.5_ averages (μg/m^3^) for 187 U.S. counties, 2000–2005.

**Figure 7:**
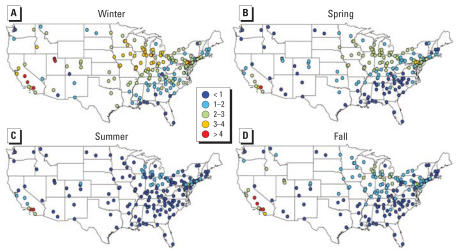
Seasonal nitrate PM_2.5_ averages (μg/m^3^) for 187 U.S. counties, 2000–2005.

Figures 4–7 map yearly and seasonal averages for the SO_4_^2−^ and NO_3_^−^ components. SO_4_^2−^ PM_2.5_ displays a strong east/west pattern (Figure 4). In the eastern United States, the SO_4_^2−^ component of PM_2.5_ typically peaks during summer (Figure 5). The NO_3_^−^component of PM_2.5_ shows a somewhat inverse pattern, with higher concentrations on the west coast, primarily in California (Figure 6). NO_3_^−^ PM_2.5_ also exhibits a north/south pattern, with higher levels in parts of the Northeast and decreasing levels towards the Southeast. This north/south gradient remains throughout all seasons (Figure 7), and highest concentrations in the eastern United States occur in winter. The western United States has the highest nitrate PM_2.5_ concentrations during winter and autumn.

The Supplemental Material (Figures S2–S11; http://www.ehponline.org/docs/2007/9621/suppl.pdf) provides maps of yearly and seasonal averages for other key components: Na^+^, Si, EC, NH_4_^+^, and OCM. Sodium ion PM_2.5_ levels are higher in coastal regions, relating to sea salt (Figure S2), and do not exhibit a strong seasonal pattern outside of a moderate trend in the western United States (Figure S3). The highest overall Si levels were noted in Texas (Figure S4), with high concentrations in other areas by season (Figure S5). EC PM_2.5_ showed spatial and temporal patterns similar to those of NO_3_^−^, without the north/south gradient in the eastern United States (Figures S6 and S7). Both EC and NO_3_^−^ PM_2.5_ were higher in California, and peaked in winter and autumn. Because ammonium (NH_4_^+^) is commonly observed in the forms of ammonium nitrate or ammonium sulfate, the ammonium component of PM_2.5_ is correlated with SO_4_^2−^ and NO_3_^−^ components and consequently exhibits a mix of those components’ spatial and temporal patterns for yearly averages (Figure S8) and seasonal averages (Figure S9). OCM is higher on the west coast (Figures S10 and S11).

Table 2 provides correlations among yearly and seasonal averages for these seven components and all other components for which the correlation coefficient reaches ≥ 0.5. Additional correlations among PM_2.5_ components are provided in the Supplemental Material (Table S4; http://www.ehponline.org/docs/2007/9621/suppl.pdf). These tables were created by first calculating the yearly and seasonal averages in each county for each component, and then calculating the correlation coefficients. NH_4_^+^ was most strongly correlated with SO_4_^2−^ and NO_3_^−^, with a stronger relationship with SO_4_^2−^ in summer (0.88) and NO_3_^−^ in winter (0.86). EC concentrations covary with Fe concentrations in all seasons; with OCM in winter, summer, and autumn; and Ti in winter and autumn. The concentrations of OCM are correlated with the levels of K in winter and Ti in autumn. Si concentrations are associated with those of crustal elements including Ca. Na^+^ concentrations are most closely associated with levels of Cl, but less so in winter. The strongest correlations for SO_4_^2−^ are with NH_4_^+^.

Table 3 provides data on the correlations between day-to-day variations of the key component concentrations and of the total PM_2.5_ mass for nationwide, eastern U.S., and western U.S. regions. Additional correlations are provided in the Supplemental Material in Table S5 (http://www.ehponline.org/docs/2007/9621/suppl.pdf). Components with the greatest contributions to total PM_2.5_ mass also had the strongest temporal correlations with PM_2.5_ total mass. The components typically co-varied with PM_2.5_ total mass when they reached peak concentrations, such as summer for SO_4_^2−^ and winter for NO_3_^−^. Of the components not listed in Table 3, K was correlated with total PM_2.5_ in winter (0.52) and Br in autumn (0.63), spring (0.55), and for yearly averages (0.56).

Using the seasonally detrended data for PM_2.5_ mass and each component, on average across the 180 counties with ≥ 1 year of data, the following components were found to have strong day-to-day variation with PM_2.5_ total mass: NH_4_+ (average correlation 0.84); SO_4_^2−^(0.78); OCM (0.68); NO_3_^−^ (0.51); Br (0.51); and EC (0.51). The relationship between daily PM_2.5_ and component concentrations varied by county. For these six components (NH_4_^+^, SO_4_^2−^, OCM, NO_3_^−^, Br, and EC), the percentages of counties with correlation coefficients > 0.6 were 95, 90, 81, 34, 33, and 22%, respectively.

We applied an alternative method of adjustment to calculate OCM, discussed previously, using blank filter values specific to the year and type of sampler (Frank NH, unpublished data). The alternative measure, designated OCM2, provided comparable results to our original OCM measure. The correlation coefficient between OCM and OCM2 was 0.99 on average across all counties (range, 0.97–1.00). Both measures of organic carbon matter (OCM and OCM2) had similar values for yearly and seasonal concentrations (Supplemental Material, Table S6; http://www.ehponline.org/docs/2007/9621/suppl.pdf), the percentage of PM_2.5_ total mass comprised of OCM by year or season (Table S7), and the correlation between OCM and PM_2.5_ total mass, NH_4_^+^, EC, NO_3_^−^, Si, Na^+^, or SO_4_^2−^, by year or season (Table S8).

## Discussion

The PM_2.5_ mixture varies strongly by region and by season, and the degree of spatial and temporal variability differs by component, which has implications for epidemiologic research on PM_2.5_ characteristics. National studies have already demonstrated that the estimated short-term effects of PM_10_ on mortality (Dominici et al. 2003; Peng et al. 2005) and of PM_2.5_ on hospital admissions (Dominici et al. 2006) vary by season and by region, with the highest effect estimates for mortality and hospital admissions in the northeastern United States during summer. These regional and temporal differences may reflect variation in the PM_2.5_ mixture and its sources.

These findings indicate the complexity of interpreting regional differences in the effect of PM_2.5_ and of designing studies directed at characterizing effects of particular components. Because of variations in the PM_2.5_ mixture, the risk associated with a particular component is assessed against a continually varying background of other pollutants. Our descriptive analyses of the new data on PM_2.5_ components illustrate the challenge of testing hypotheses around specific components such as explaining observed seasonal and regional variation in the effect of PM_2.5_ (Peng et al. 2005).

Many techniques are available to determine the sources of PM_2.5_ components, including factor analysis, Gaussian plume modeling, and backward trajectory modeling; each has its own set of advantages and limitations (Hopke et al. 2006; Ito et al. 2006; Laden et al. 2000; Lapina and Paterson 2004; Mar et al. 2006; Paatero et al. 2003; Thurston et al. 2005). Methodologies that assign specific components or sets of components to sources face the challenge that any individual PM_2.5_ component comes from a variety of sources. Table S9 in the Supplemental Material (http://www.ehponline.org/docs/2007/9621/suppl.pdf) lists some sources of the seven key components identified. Approaches to linking components to specific sources may be more suitable for localized studies in which dominant sources can be identified, for example in cases where detailed knowledge is available regarding sources for the region.

For studies based on national data or large regions, multiple sources of each component may complicate such efforts (Table S9). For example, Selenium (Se) and SO_4_^2−^ had an overall correlation of 0.47 in this data set, and both can result from combustion of coal, oil, or biomass. However, Se emissions also come from smelters and coke production, and SO_4_^2−^ emissions result from motor vehicles, incineration, electronics manufacturing, steel mills, and other sources. EC and Fe both come from traffic emissions, vegetative burning, oil combustion, and casting processes. Na^+^ and Cl co-vary because of a common origin in oceans; Na^+^, however, also comes from other sources, and Cl can result from combustion emissions from cooking, coal, automobiles, vegetation burning, and incinerators.

These findings suggest that the new data on PM_2.5_ components may not lead to satisfactory, definitive source apportionment for national studies. In localized settings, source apportionment and related methodologies are more appropriate. Correlated concentrations and multiple sources complicate the identification of individual effects of various PM_2.5_ components on a national scale. For example, SO_4_^2−^ concentrations are associated with NH_4_^+^ and Se concentrations. Therefore, a study identifying PM_2.5_ sulfate as associated with adverse health impacts may be detecting effects of co-varying pollutants (e.g., Se, ammonium sulfate, or other components with similar sources for that region). Because every component in the data set has multiple and shared sources, no pairs of the components are perfectly correlated; the highest correlation of yearly averages (0.998) was for cerium and lanthanum. Therefore, methods using a single component or set of components as source surrogates [e.g., SO_4_^2−^ and Se for coal combustion, vanadium (V) for oil combustion, EC for traf-fic] for national studies can be affected by some misclassification of contributing sources that might also vary by region and season. In local studies with less spatial heterogeneity of source profiles, methods such as source apportionment are more likely to be successful. Further, in studies of smaller regions, additional PM_2.5_ chemical component data may be available, including concentrations of ammonium sulfate, rather than NH_4_^+^ and SO_4_^2−^ separately.

We found that of the 52 components considered, only seven contributed ≥ 1% to total PM_2.5_ mass for the yearly average or any seasonal average. We also found that several of these seven components are also correlated with day-to-day variations in the PM_2.5_ total mass. Results indicate that the strongest correlations with PM_2.5_ total mass are NH_4_+ (yearly, all seasons), OCM (especially winter), NO_3_^−^(winter), and SO_4_^2−^ (yearly, spring, autumn, and summer), with particularly strong correlations for NH_4_+ or SO_4_^2−^ in summer.

The observed health risks of PM_2.5_ could be a function of the key components identified above; however, other explanations are also possible. These alternative scenarios include a component contributing < 1% to total PM_2.5_ mass, but with concentrations below detection limits that co-vary with PM_2.5_ total mass, or a component or set of components that co-vary with the identified key components. To gather evidence toward these alternative explanations, we evaluated which components co-varied with the seven key components as shown in Table 2, andwhich components co-varied with PM_2.5_ detrended data, which identified six of the key components.

Limitations of these data and analyses include measurement error and detection limits, which may hinder identification of relationships among components or a component’s contribution to PM_2.5_ total mass (Flanagan et al. 2006; Frank 2006; Schwab et al. 2006). These limitations may affect some chemical components more than others because of differing instrument abilities for detection and measurement. For example, the ratio of *OCM* to *OC_m_*−*OC_b_* [i.e., *k* (Equation 1)] can vary by site and season (Bae et al. 2006, Turpin and Lim 2001) although such specific adjustments to *OC_m_* are currently not possible. In particular, *k* may be higher in rural settings than in urban settings. Even the levels of PM_2.5_ total mass are subject to measurement error. Because not all possible PM_2.5_ components were measured, the sum of measured PM_2.5_ component concentrations was generally but not universally less than the total PM_2.5_ mass. However, the sum of components can exceed PM_2.5_ total mass because of negative artifacts such as loss of ammonium nitrate and other semivolatile organics (Frank 2006).

Because of these limitations, health risks could be associated with the true concentrations of a component or set of components that co-varies with PM_2.5_ total mass, even if measured concentrations in this data set do not co-vary with PM_2.5_ total mass because of measurement error. Further, we did not investigate the possibility that observed PM_2.5_ health effects could result from a set of components with a collective concentration that co-varies with PM_2.5_ total mass, although individual component concentrations do not.

## Figures and Tables

**Table 1:** Yearly, summer, and winter concentrations for the PM_2.5_ components, on average across 187 U.S. counties.

	Yearly		Summer		Winter	
	Mean ± SD	IQR (min–max)	Mean ± SD	IQR (min–max)	Mean ± SD	IQR (min–max)
Aluminum	29.2 ± 1.48	11.4 (10.2–171)	43.6 ± 2.98	27.6 (11.5–391)	17.3 ± 0.80	6.22 (2.18–71.5)
Ammonium	1,543 ± 42.6	729 (227–3,889)	1,699 ± 61.1	1,198 (121–5,028)	1,591 ± 43.4	772 (196–3,965)
Antimony	11.1 ± 0.23	2.41 (3.4–17.7)	11.2 ± 0.23	2.87 (2.96–17.6)	11.2 ± 0.23	3.24 (2.96–18.9)
Arsenic	1.70 ± 0.04	0.58 (0.6–4.46)	1.7 ± 0.06	0.62 (0.57–7.89)	1.65 ± 0.04	0.53 (0.50–4.07)
Barium	24.2 ± 0.48	7.34 (9.98–39.4)	24.9 ± 0.52	8.73 (9.11–41.1)	23.5 ± 0.53	8.23 (7.51–41.0)
Bromine	3.14 ± 0.09	1.10 (1.34–13.9)	2.61 ± 0.07	0.91 (1.11–8.82)	3.71 ± 0.14	1.68 (1.32–22.3)
Cadmium	5.51 ± 0.11	0.71 (2.16–7.18)	5.4 ± 0.11	0.73 (2.23–9.50)	5.62 ± 0.11	0.92 (2.11–8.03)
Calcium	57.0 ± 3.57	36.5 (12.4–450)	63.3 ± 3.68	39.1 (14.3–428)	45.6 ± 3.42	30.4 (9.19–478)
Cerium	29.5 ± 0.6	9.81 (8.86–44.5)	30.4 ± 0.65	12.1 (9.6–51.6)	27.6 ± 0.62	9.26 (4.87–45.2)
Cesium	13.4 ± 0.27	3.41 (4.03–19.1)	13.8 ± 0.29	4.1 (5.11–22.8)	12.8 ± 0.29	3.61 (2.53–22.4)
Chlorine	24.8 ± 2.32	21.3 (3.25–300)	14.0 ± 2.16	4.91 (2.73–322)	44.4 ± 4.02	47.2 (4.47–414)
Chromium	2.03 ± 0.12	0.92 (0.42–19.13)	2.04 ± 0.1	1.06 (0.45–11.4)	2.16 ± 0.23	0.97 (0.38–39.5)
Cobalt	0.71 ± 0.01	0.06 (0.28–1.41)	0.71 ± 0.01	0.07 (0.28–1.4)	0.72 ± 0.02	0.06 (0.28–1.49)
Copper	3.98 ± 0.22	2.46 (1.00–23.5)	4.54 ± 0.28	2.71 (1.13–36.8)	4.16 ± 0.23	2.74 (0.64–24.6)
EC	629 ± 19.6	283 (166–1742)	540 ± 18.5	264 (143.6–1,899)	721 ± 27.3	406 (156.3– 2,126)
Europium	4.56 ± 0.09	1.21 (1.72–7.75)	4.64 ± 0.1	1.14 (1.74–10.9)	4.40 ± 0.1	1.09 (1.13–8.97)
Gallium	1.63 ± 0.03	0.22 (0.59–2.25)	1.66 ± 0.03	0.33 (0.55–2.35)	1.60 ± 0.03	0.23 (0.59–2.19)
Gold	2.74 ± 0.06	0.44 (1.07–3.87)	2.92 ± 0.06	0.71 (1.01–4.39)	2.66 ± 0.06	0.50 (0.89–3.66)
Hafnium	11.3 ± 0.22	1.19 (4.29–14.1)	11.3 ± 0.22	1.93 (3.92–16.3)	11.5 ± 0.22	1.61 (4.87–15.4)
Indium	6.27 ± 0.12	0.91 (2.33–8.22)	6.29 ± 0.13	0.97 (2.43–10.6)	6.38 ± 0.13	1.17 (2.26–9.11)
Iridium	3.16 ± 0.06	0.44 (1.07–4.57)	3.29 ± 0.07	0.79 (1.01–4.56)	3.04 ± 0.06	0.62 (1.03–4.25)
Iron	85.7 ± 3.91	44.4 (15.39–437)	93.0 ± 4.09	39.9 (18.5–455)	77.7 ± 4.59	44.3 (11.0–635)
Lanthanum	23.3 ± 0.47	7.92 (6.79–35.1)	23.9 ± 0.51	9.5 (8.91–42.6)	22.1 ± 0.49	7.4 (3.7–34.7)
Lead	4.89 ± 0.21	1.82 (1.63–23.6)	4.74 ± 0.32	1.81 (1.33–51.0)	5.01 ± 0.19	2.21 (1.5–22.4)
Magnesium	15.3 ± 0.43	3.28 (7.17–67.6)	18.6 ± 0.60	6.83 (4.46–76.3)	12.6 ± 0.39	3.12 (3.69–62.62)
Manganese	3.00 ± 0.22	1.41 (0.71–32.2)	2.84 ± 0.18	1.32 (0.72–22.3)	3.08 ± 0.27	1.53 (0.77–39.8)
Mercury	2.39 ± 0.04	0.28 (0.91–3.94)	2.34 ± 0.04	0.38 (0.88–3.31)	2.42 ± 0.05	0.44 (0.88–5.01)
Molybdenum	3.1 ± 0.06	0.49 (1.14–6.21)	3.14 ± 0.07	0.61 (1.03–8.61)	3.18 ± 0.07	0.52 (0.96–5.79)
Nickel	1.85 ± 0.17	0.86 (0.33–20.2)	1.67 ± 0.12	0.82 (0.33–13.9)	2.4 ± 0.33	1.02 (0.3–31.3)
Niobium	1.98 ± 0.04	0.18 (0.78–2.48)	2.00 ± 0.04	0.32 (0.74–2.59)	1.95 ± 0.04	0.21 (0.74–2.45)
Nitrate	1,733 ± 84.9	1,298 (327–10,017)	836 ± 76.3	567 (119–11,814)	2,990 ± 122	2,059 (657–11,451)
OCM	3,823 ± 100.9	1,373 (967–12,120)	4,413 ± 77.1	1,432 (1,910–7,604)	3,995 ± 185	2,150 (152–24,332)
Phosphorus	4.80 ± 0.09	0.82 (1.26–7.9)	5.07 ± 0.12	1.46 (1.26–11.8)	4.49 ± 0.11	0.73 (1.26–15.5)
Potassium	72.9 ± 2.41	27.4 (23.1–275)	85.4 ± 3.13	43.8 (22.9–309)	73.2 ± 2.64	31.1 (20.9–274)
Rubidium	0.99 ± 0.02	0.07 (0.41–1.33)	1.00 ± 0.02	0.16 (0.41–1.27)	0.96 ± 0.02	0.13 (0.32–1.40)
Samarium	3.00 ± 0.05	0.3 (1.24–5.48)	3.17 ± 0.07	0.5 (1.24–11.9)	2.82 ± 0.05	0.38 (1.09–4.56)
Scandium	2.10 ± 0.06	0.67 (0.49–5.39)	1.76 ± 0.06	0.69 (0.38–5.52)	2.38 ± 0.07	0.82 (0.48–6.01)
Selenium	1.62 ± 0.05	0.44 (0.51–7.49)	1.59 ± 0.05	0.46 (0.53–7.11)	1.73 ± 0.05	0.68 (0.52–5.91)
Silicon	105 ± 4.70	49.6 (35.1–454)	147 ± 7.10	87.0 (30.5–795)	65.0 ± 3.38	25.1 (19.5–352)
Silver	5.06 ± 0.1	0.36 (2.11–6.44)	5.02 ± 0.10	0.66 (2.05–7.10)	5.00 ± 0.10	0.66 (1.94–6.76)
Sodium ion	128 ± 5.10	58.15 (37.2–509)	130 ± 6.30	60.3 (24.8–620)	142 ± 4.70	70.1 (45.8–606)
Strontium	1.49 ± 0.03	0.23 (0.57–4.11)	1.77 ± 0.05	0.53 (0.56–6.22)	1.41 ± 0.04	0.24 (0.51–4.82)
Sulfate	3,698 ± 102.4	2,020 (658–6,604)	5,256 ± 172	3,527 (523–9,304)	2,524 ± 62	1,026 (446–5,925)
Tantalum	8.67 ± 0.19	3.26 (2.43–14.8)	9.06 ± 0.22	3.87 (2.85–18.4)	8.07 ± 0.19	2.54 (1.73–15.3)
Terbium	3.85 ± 0.11	0.64 (1.48–17.6)	3.93 ± 0.13	0.86 (1.37–21.5)	3.72 ± 0.11	0.78 (1.36–19.4)
Tin	10.18 ± 0.19	1.15 (4.34–15.7)	10.49 ± 0.2	1.99 (3.86–14.8)	9.91 ± 0.19	1.69 (3.92–13.4)
Titanium	5.33 ± 0.16	1.87 (1.69–16.2)	6.96 ± 0.23	2.82 (2.25–22.3)	4.18 ± 0.17	1.55 (1.18–18.5)
Tungsten	2.15 ± 0.12	0.81 (0.62–10.6)	2.17 ± 0.13	0.65 (0.6–12.4)	2.31 ± 0.13	1.11 (0.54–9.80)
Vanadium	5.64 ± 0.11	1.23 (1.96–7.4)	5.76 ± 0.12	1.42 (2.01–8.01)	5.51 ± 0.11	1.23 (1.79–8.11)
Yttrium	1.40 ± 0.03	0.14 (0.56– 1.71)	1.42 ± 0.03	0.23 (0.56–1.88)	1.38 ± 0.03	0.13 (0.47–1.84)
Zinc	14.0 ± 0.98	7.67 (1.59–130)	11.21 ± 1.00	7.39 (1.29–144)	17.2 ± 0.97	9.13 (1.84–125)
Zirconium	1.9 ± 0.04	0.23 (0.74–3.03)	1.94 ± 0.04	0.32 (0.74–4.71)	1.86 ± 0.04	0.26 (0.72–3.25)
PM_2.5_ (μg/m^3^)	14.0 ± 0.22	4.09 (5.04–26.0)	16.19 ± 0.34	7.29 (5.59–28.5)	13.9 ± 0.27	3.5 (5.06–32.8)

Abbreviations: IQR, interquartile range; min–max, minimum to maximum. Units are in ng/m^3^ except for PM_2.5_ total mass, which is in μg/m^3^.

**Table 2:** Correlations among selected PM_2.5_ chemical components, on average across 187 U.S. counties.

	EC	OCM	Si	Na^+^	SO_4_^2−^	NO_3_^−^	Br	Ca	Cl	Cu	Fe	Mg	K	Se	Ti
Yearly averages															
NH_4_^+^			−		0.72	0.64	+							+	
EC		0.59	+	+		+	+	+	0.52	+	0.65	+	+		0.57
OCM			+							+	+	+	+		+
Si					−			0.68			+	+	+		0.78
Na^+^									0.63				+		+
SO_4_^2−^							+	−				−		+	−
NO_3_^−^							+			+	+				
Winter averages															
NH_4_^+^					+	0.86	+		+					+	
EC		0.73	0.57					+	+	0.50	0.62	+	+		0.66
OCM			+							+	+		0.64		+
Si					−			0.73		+	0.56				0.71
Na^+^									+				+		
SO_4_^2−^							+							+	−
NO_3_^−^									+						
Spring averages															
NH_4_^+^	+		−		0.70	0.74	+							+	
EC		0.51					+	+	+	+	0.60	+			+
OCM					+		+				+		+		+
Si					−			0.76			+	+	+		0.81
Na^+^									0.73						
SO_4_^2−^							0.54	−						+	
NO_3_^−^															
Summer averages															
NH_4_^+^	+	+	−		0.88	0.53	+	−						+	
EC		+				+	+		+	+	0.57		+	+	+
OCM					+	+	+						+		
Si					−			0.57	+		0.54	+			0.84
Na^+^						+			0.63			+			+
SO_4_^2−^							+	−				−		0.50	−
NO_3_^−^							+						+		
Autumn averages															
NH_4_^+^	+				0.66	0.62	0.59				+			+	
EC		0.57	+			+	+	+	+	+	0.69	+	+		0.62
OCM			+	+		+	+			+	+		+		0.51
Si					−	+		0.65		+	+	0.51	+		0.75
Na^+^						+			0.58				+		
SO_4_^2−^							+							+	
NO_3_^−^							+			+	+				+

Correlations < 0.25 are not shown; 0.25 to 0.50 are depicted as +; −0.50 to −0.25 are depicted as −; and > 0.50 are shown as a numerical value.

**Table 3:** Correlations of selected PM_2.5_ chemical components with PM_2.5_ total mass for the United States and eastern and western regions.

	Yearly	Winter	Spring	Summer	Autumn
U.S.					
NH_4_^+^	0.83	0.66	0.82	0.90	0.82
EC	+	0.53	+	+	+
OCM	0.52	0.70	0.61	0.56	0.63
Si				−	
Na^+^	0.72		0.79	0.94	0.63
SO_4_^2−^	+	0.66	+	+	+
NO_3_^−^					
Eastern U.S.					
NH_4_^−^	0.75	0.76	0.66	0.84	0.73
EC	0.54	0.50	0.53	+	+
OCM	0.69	0.59	0.70	0.63	0.73
Si		+	+		+
Na^+^					
SO_4_^2−^	0.84	0.57	0.76	0.94	0.87
NO_3_^−^		0.52		+	
Western U.S.					
NH_4_^+^	0.88	0.72	0.89	0.96	0.89
EC	0.65	0.52	0.72	0.62	0.69
OC	0.71	0.76	0.68	0.62	0.78
Si					
Na^+^	0.50		+	0.54	0.54
SO_4_^2−^	0.68	+	0.83	0.86	0.67
NO_3_^−^	0.91	0.75	0.90	0.96	0.91

Correlations < 0.25 are not shown; 0.25 to 0.50 are depicted as +; −0.50 to −0.25 are depicted as −; and > 0.50 are shown as a numerical value.
